# Tailoring structural and magnetic properties of Mn_3−*x*_Fe_*x*_Ga alloys towards multifunctional applications

**DOI:** 10.1107/S205225251801326X

**Published:** 2018-10-17

**Authors:** Z.H. Liu, Z. J. Tang, J. G. Tan, Y. J. Zhang, Z. G. Wu, X. T. Wang, G. D. Liu, X. Q. Ma

**Affiliations:** a Department of Physics, University of Science and Technology Beijing, Beijing 100083, People’s Republic of China; b School of Civil Engineering, Guangzhou University, Guangzhou 510006, People’s Republic of China; c School of Physical Science and Technology, Southwest University, Chongqing 400715, People’s Republic of China; d School of Material Science and Engineering, Hebei University of Technology, Tianjin 300130, People’s Republic of China

**Keywords:** Mn–Fe–Ga alloys, tetragonal Heusler alloys, multiple structural materials

## Abstract

This study investigated the structural and magnetic properties of Mn_3−*x*_Fe_*x*_Ga alloys (*x* = 0, 0.2, 0.4, 0.6, 0.8, 1) under different heat-treatment conditions. A tetragonal structure was observed in samples that were heat treated at 623 K for three days followed by quenching in ice water. These tetragonal alloys present large coercive fields in the range 0.8–5 kOe and low saturation magnetization, and have great potential for application in spin-transfer torque-based devices. A hexagonal structure was observed in samples subjected to heat treatment at 883 K for three days following quenching in ice water. A moderate decrease in the coercive field has been observed for the hexagonal alloys compared with those with a tetragonal structure. However, the Mn_3−*x*_Fe_*x*_Ga alloys with a hexagonal structure exhibit other attractive magnetic properties, including collinear and non-collinear magnetic properties, holding high promise for technological applications. A face-centred-cubic (f.c.c.) structure was observed when subjected to annealing at 1073 K for three days followed by quenching in ice water. In contrast to the tetragonal and hexagonal structures, all f.c.c. alloys exhibit antiferromagnetic behaviour. This versatile material can display a wide range of multi-functionalities attributed to its tuneable crystal structure. This investigation will guide the design of multiple structures of these materials in order to utilise the wide functionalities for practical applications.

## Abstract 2

The crystal structures and magnetic properties of Mn_3−*x*_Fe_*x*_Ga alloys (*x* = 0, 0.2, 0.4, 0.6, 0.8, 1) can be extensively tailored by different heat-treatment conditions. This investigation sheds light on tuning the crystal structures of these materials *via* heat treatment towards wide functionalities for technological applications.

## 1. Introduction   

Heusler compounds have attracted a large amount of attention since they exhibit rich functional properties, including the shape-memory effect, superconductivity, half metallicity, magnetocaloric effect and spin-gapless semiconductivity (Liu *et al.*, 2003 ▸; Klimczuk *et al.*, 2012 ▸; Jamer *et al.*, 2017 ▸; Li *et al.*, 2018 ▸; Wang *et al.*, 2017 ▸). One of the most recently studied binary Heusler alloys, Mn_3_Ga, shows the presence of multiple phases with interesting structural, magnetic and electron-transport properties, with the potential for use in spintronic and other magnetic applications (Chadov *et al.*, 2015 ▸; Khmelevskyi *et al.*, 2016*a* ▸; Kharel *et al.*, 2014 ▸). The alloy is reported to have diverse crystal structures including tetragonal, hexagonal and face-centred cubic (f.c.c.) when subjected to different heat treatments (Kharel *et al.*, 2014 ▸; Winterlik *et al.*, 2008 ▸; Khmelevskyi *et al.*, 2016*b* ▸). Hexagonal Mn_3_Ga (70–74at.% Mn) has a D0_19_-type structure with the space group *P*63/*mmc* (Niida *et al.*, 1983 ▸), which favours the formation of a triangular antiferromagnetic spin structure, giving rise to a large anomalous Hall effect at room temperature and the topological Hall effect (THE) at temperatures below 100 K (Liu *et al.*, 2017 ▸). THE, caused by the non-vanishing Berry phase in a non-collinear spin arrangment antiferromagnet, has great prospects in fundamental science and applications. Tetragonal Mn_3_Ga is ferrimagnetic with low magnetization and a high Curie temperature, having potential applications in spin-transfer torque (STT) devices (Chadov *et al.*, 2015 ▸; Kurt *et al.*, 2011 ▸). The Mn_3_Ga alloy with an f.c.c. structure was found to exhibit an antiferromagnetic spin order at room temperature and Kondo-like electron transport at low temperatures (Kharel *et al.*, 2014 ▸).

Among these, tetragonal Mn_3_Ga was most extensively investigated for its potential applications in STT devices. Attempts have been made to use the elements Co, Fe, Ni and Pt as dopants for the partial substitution of Mn in order to explore more promising magnetic properties (Winterlik *et al.*, 2012 ▸; Felser *et al.*, 2013 ▸; Nayak *et al.*, 2015 ▸), especially when aiming for a large coercive field. Felser *et al.* reported that the Mn_2_FeGa alloy with an f.c.c. structure is almost in a compensated magnetic state (Nayak *et al.*, 2015 ▸). Adjustments to the compositions of the alloys have resulted in the emergence of exchange-bias effects in f.c.c. Mn_1.5_Fe_1.5_Ga and Mn_1.8_Fe_1.2_Ga alloys (Nayak *et al.*, 2015 ▸). In previous work, we presented our experimental observation of a D0_19_-type hexagonal structure and an accompanying large exchange-bias effect in a polycrystalline Mn_2_FeGa alloy when subjected to suitable heat treatment (Liu *et al.*, 2016 ▸).

Multiple-crystal-structure materials hold high promise of diverse magnetic properties, which has enabled them to become a ‘hot’ research subject among multifunctional materials. A key issue in realizing the multi-functionalities of these materials is finding an easy way to artificially regulate the alloy structure in order to meet the performance for technological applications. To solve this problem, understanding the structural stability of the alloy is very important. The phase equilibrium in the Mn-rich portion of the Mn–Ga binary system has been experimentally determined (Krén & Kádár, 1970 ▸; Tsuboya & Sugihara, 1963 ▸; Masumoto *et al.*, 1978 ▸; Minakuchi *et al.*, 2012 ▸). However, the phase stability of Mn–Ga-based Mn_3−*x*_Fe_*x*_Ga has not yet been investigated in detail. In this work, we conducted a systematic study of the Mn_3−*x*_Fe_*x*_Ga alloy (*x* = 0, 0.2, 0.4, 0.6, 0.8, 1), focusing on the thermal stability, various structures and related magnetic properties, which will provide an approach for multifunctional materials design.

## 2. Experimental methods   

Polycrystalline samples of Mn_3−*x*_Fe_*x*_Ga (*x* = 0, 0.2, 0.4, 0.6, 0.8, 1) with nominal compositions were smelted from elemental Mn, Fe and Ga of 99.99% purity in an arc furnace under the protection of an argon atmosphere. The alloys were allowed to solidify in the furnace and were remelted in several heating cycles to ensure composition homogeneity. The ingots were heat treated at 623, 883 and 1073 K for 3 d under vacuum followed by quenching in ice water. The samples were cut into slices to measure the crystal structure, which was characterized by X-ray diffraction (XRD) using a Rigaku SmartLab 3 instrument with Cu *K*α radiation. Differential scanning calorimetry (DSC) analysis was conducted using a NETZSCH STA449F3 with a simultaneous thermal analyzer having a heating/cooling rate of 10 K min^−1^. The magnetic measurements were performed using a Physical Properties Measurement System (PPMS-9, Quantum Design, Inc.). The Hall resistances were measured using a standard four-probe method with PPMS.

First-principle band calculations were carried out using the plane-wave pseudo-potential method with the CASTEP code in the framework of density functional theory. The GGA of PW91 was used to describe the exchange correlation potential. A Monkhorst–Pack grid (15 × 15 × 15) with 120 summarized *k* points for the cubic structure and 13 × 13 × 12 for the tetragonal structure was used for the Brillouin zone with a cut-off energy of 500 eV and a self-consistent field tolerance of 10^−6^ eV. The quality of the *k*-point separation for the band-structure calculation is 0.015 Å^−1^.

## 3. Results and discussion   

**Figure 1:**
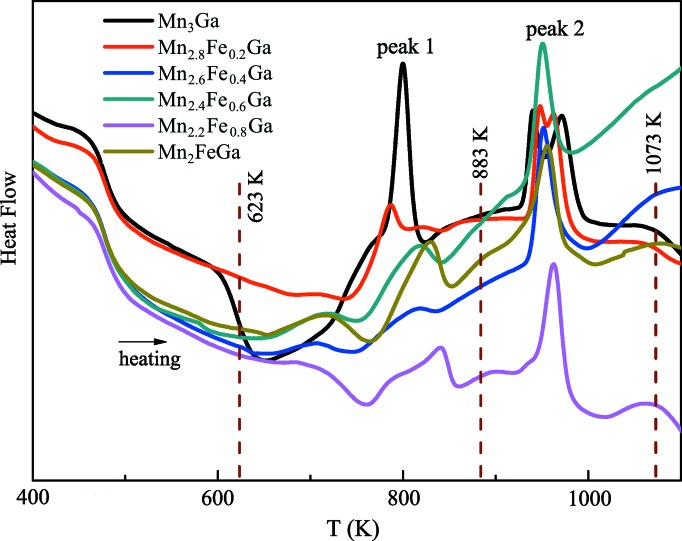
DSC measurements of the as-cast ingot samples of Mn_3−*x*_Fe_*x*_Ga (*x* = 0, 0.2, 0.4, 0.6, 0.8, 1) during the heating process.

Fig. 1 ▸ shows the DSC measurements of the as-cast ingot samples of Mn_3−*x*_Fe_*x*_Ga (*x* = 0, 0.2, 0.4, 0.6, 0.8, 1) during heating. The phase evolution is clearly observable from the DSC curves. The endothermic transformation peaks, namely peak 1 and peak 2, are evident from 400 to 1100 K, suggesting phase development during the heating process. It should be noted that no exothermic peak was observed during cooling (not shown). Based on the temperature ranges of the endothermic peaks, three thermal-treatment temperatures, 623, 883 and 1073 K, were selected for phase identification.

**Figure 2:**
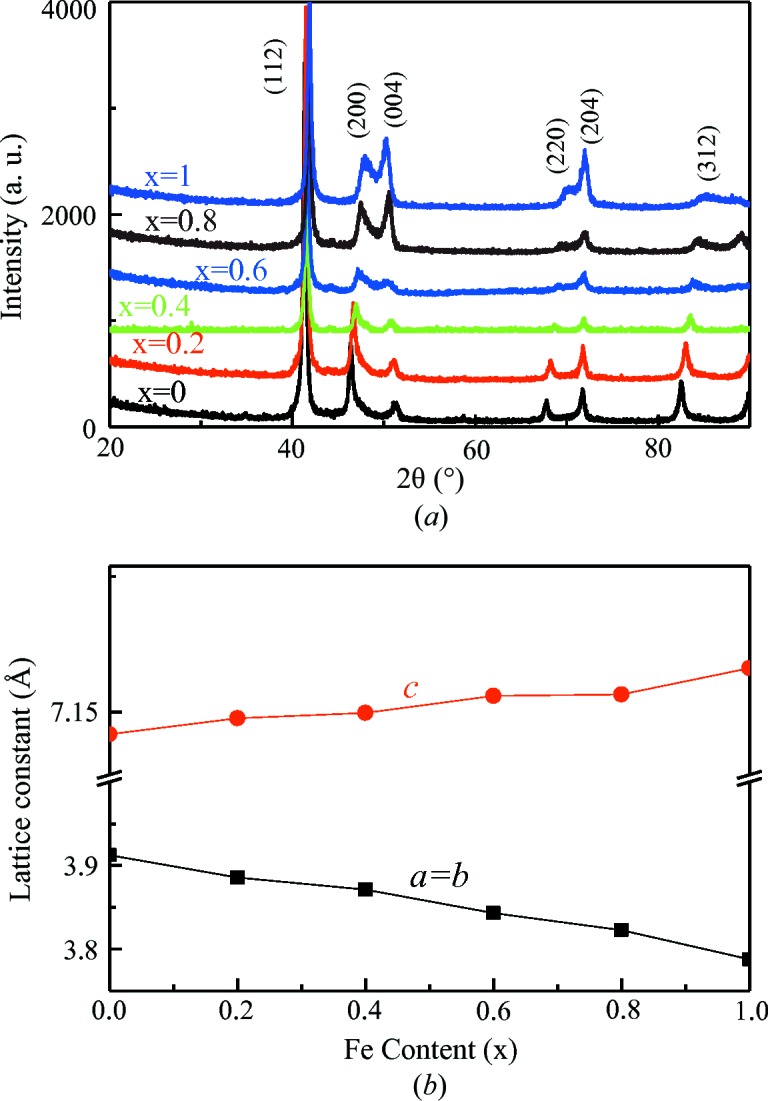
(*a*) XRD spectra measured at room temperature for Mn_3−*x*_Fe_*x*_Ga samples heat treated at 623 K; (*b*) effects of Fe substitution with Mn on the lattice parameters.

Fig. 2 ▸(*a*) shows XRD spectra measured at room temperature for Mn_3−*x*_Fe_*x*_Ga (*x* = 0, 0.2, 0.4, 0.6, 0.8, 1) samples heat treated at 623 K. All samples showed a tetragonal (D0_22_) structure. This is different from the case of Mn_3−*x*_Co_*x*_Ga (Felser *et al.*, 2013 ▸), in which the sample had a tetragonal structure only when Co concentrations were low (*x* < 0.5), but turned into a cubic structure for higher Co concentrations(*x* > 0.5). Fig. 2 ▸(*b*) shows the effect of Fe content on the lattice parameters of alloys at room temperature. The lattice constants are *a* = *b* = 3.9123, *c* = 7.0971 Å and *a* = *b* = 3.7879, *c* = 7.2557 Å for Mn_3_Ga and Mn_2_FeGa, respectively. It is observed that *a* and *b* axes contract while the *c* axis expands with increasing Fe content, resulting in a near-linear decrease in cell volume from 108.629 Å^3^ in Mn_3_Ga to 104.106 Å^3^ in Mn_2_FeGa. The *c*/*a* ratio increases gradually from 1.814 to 1.915 as *x* increases from 0 to 1.

**Figure 3:**
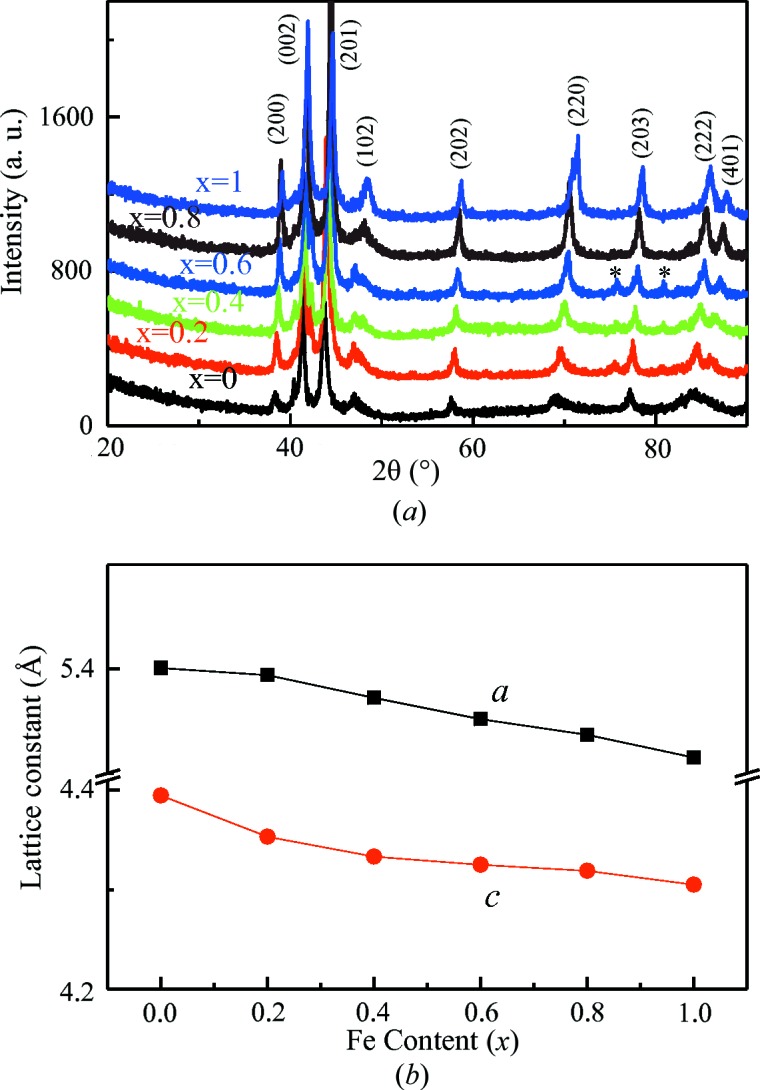
(*a*) XRD spectra measured at room temperature for Mn_3−*x*_Fe_*x*_Ga samples heat treated at 883 K; (*b*) effects of Fe substitution with Mn on the lattice parameters.

Fig. 3 ▸(*a*) shows the room temperature XRD spectra for samples heat treated at 883 K. The diffraction peaks of all the samples were indexed to be D0_19_-type (Ni_3_Sn-type) hexagonal structures (Feng *et al.*, 2006 ▸). The weaker peaks marked by a * sign may be from *α-*Mn. The lattice parameters as a function of Fe content are shown in Fig. 3 ▸(*b*). It can be clearly seen that the lattice constants *a* and *c* decrease with increasing Fe content, resulting in a decrease in the cell volume. It should be noted that although Mn_3−*x*_Fe_*x*_Ga alloys have the chemical formula of Heusler alloys, they appear to form hexagonal structures under suitable heat-treatment conditions.

**Figure 4:**
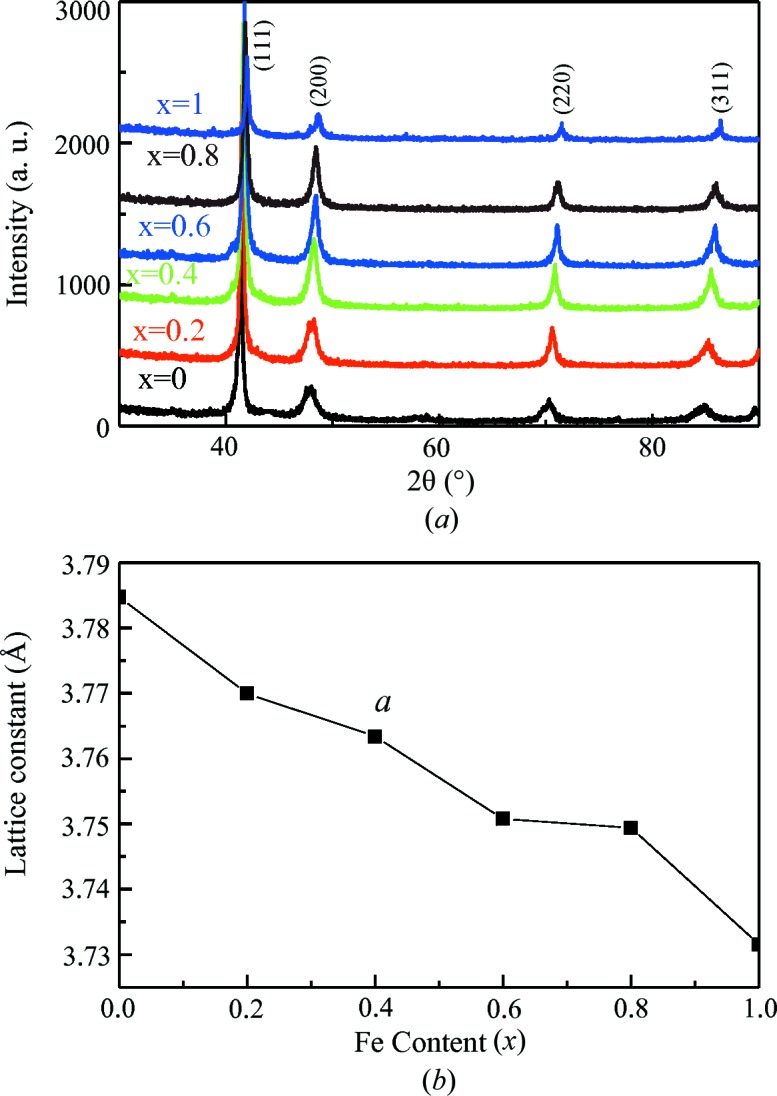
(*a*) XRD spectra measured at room temperature for Mn_3−*x*_Fe_*x*_Ga samples heat treated at 1073 K; (*b*) effects of Fe substitution with Mn on the lattice parameters.

Fig. 4 ▸(*a*) shows XRD patterns measured at room temperature for samples of Mn_3−*x*_Fe_*x*_Ga (*x* = 0, 0.2, 0.4, 0.6, 0.8, 1) heat treated at 1073 K. All the peaks have been indexed according to the standard pattern of an f.c.c. Cu_3_Au-like structure (Kharel *et al.*, 2014 ▸). The alloys with a Cu_3_Au-like f.c.c. structure are well known for forming disordered structures easily, in contrast to the common highly ordered cubic Heusler alloys. The lattice parameters decrease with increasing Fe content (see Fig. 4 ▸ *b*).

**Figure 5:**
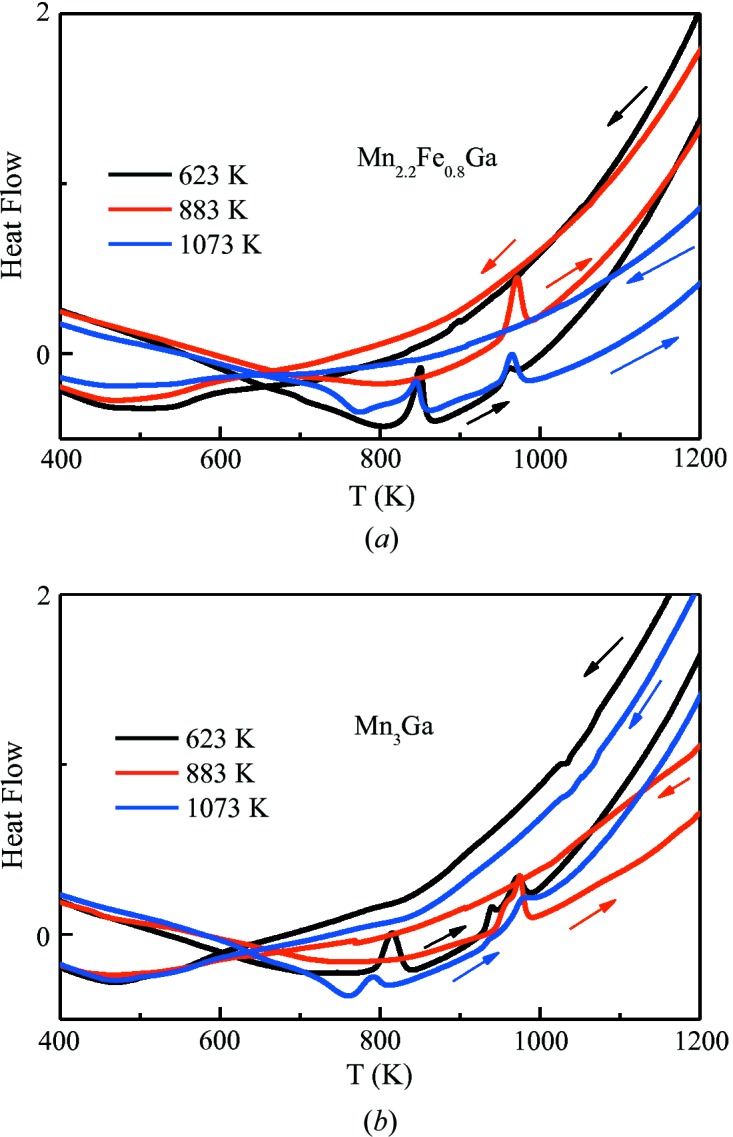
DSC measurements for (*a*) Mn_2.2_Fe_0.8_Ga and (*b*) Mn_3_Ga treated at different temperatures.

In order to further determine the phase stability, DSC measurements for samples heat treated at 623, 883 and 1073 K were carried out. We used Mn_2.2_Fe_0.8_Ga and Mn_3_Ga alloys as examples, as shown in Fig. 5 ▸. For both alloys, two endothermic transformation peaks can be clearly observed for samples treated at 623 and 1073 K during heating, and there was no exothermic peak present upon cooling. However, for samples treated at 883 K, only one endothermic peak was observed upon heating with the absence of an exothermic peak upon cooling. Samples treated at 623 K have tetragonal structures at room temperature. The samples experienced a transformation sequence of tetragonal → hexagonal → f.c.c. upon heating. The samples have hexagonal structures at room temperature after heat treatment at 883 K. The endothermic peak that appears around 970 K during heating can be attributed to the hexagonal → f.c.c. structure transformation. The samples possess f.c.c. structures at room temperature following heat treatment at 1073 K. During the heating process, they firstly underwent an f.c.c. → hexagonal transformation, followed by a hexagonal → f.c.c. transformation with further heating. We can now ask the question: why is there no exothermic peak upon cooling? It is possible that the driving force is not large enough to drive the reverse transformation, or the transformation range is too wide to identify a peak. The exact reason is not clear at this stage and further investigation is needed.

Although Mn_3_Ga (Mn_2_MnGa) and Mn_2_FeGa have the chemical formula of Heusler alloys and the elements Mn, Fe are the neighbours of Co in the periodic table, their structures are rather different from the Heusler-type Mn_2_CoGa alloys. Mn_2_CoGa can be crystallized in a cubic Heusler structure. However, Mn_3_Ga and Mn_2_FeGa have been observed experimentally to form tetragonal Heusler structures only. In order to investigate the phase stability of Heusler-type alloys of Mn_3_Ga and Mn_2_FeGa, we performed first-principles calculations for Mn_2_ *Y*Ga (*Y* = Mn, Fe, Co) alloys.

**Figure 6:**
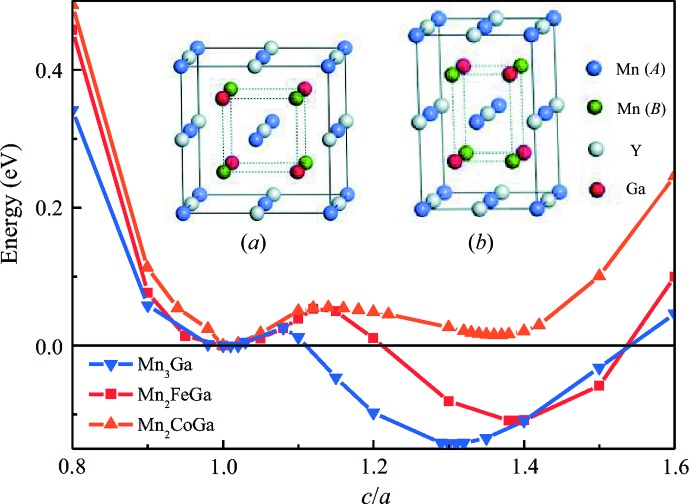
Total energy of the tetragonal structure as a function of the *c*/*a* ratio for Mn_2_ *Y*Ga alloys (*Y* = Mn, Fe, Co). Insets show schematic representations of the (*a*) cubic and (*b*) tetragonal Heusler compounds of Mn_2_ *Y*Ga.

It is assumed that Mn_2_ *Y*Ga (*Y* = Mn, Fe, Co) alloys have cubic Heusler-type structures, and they are likely to crystallize into Hg_2_CuTi-type Heusler alloys (Burch *et al.*, 1974 ▸), in which Mn atoms occupy Wyckoff sites *a* (0, 0, 0) and *b* (0.25, 0.25, 0.25) [denoted as Mn(*a*) and Mn(*b*)], *Y* occupies the Wyckoff site *c* (0.5, 0.5, 0.5) and Ga occupies site *d* (0.75, 0.75, 0.75), as shown in inset (*a*) of Fig. 6 ▸. The total energy for tetragonal distortion [see inset (*b*) of Fig. 6 ▸] of the cubic structure along the *c* axis was calculated, assuming that the volume for the equilibrium state does not change with tetragonal distortions (Sahariah *et al.*, 2012 ▸; Paul & Ghosh, 2011 ▸). The total energy of the tetragonal structure as a function of *c*/*a* is depicted in Fig. 6 ▸. It can be seen that only Mn_2_CoGa has the lowest energy at *c*/*a* = 1, suggesting that the ground state is a cubic structure for Mn_2_CoGa, which is in agreement with the experimental result; while for Mn_3_Ga and Mn_2_FeGa alloys, the energy minima are located when *c*/*a* > 1. The energy minima correspond to *c*/*a* = 1.3 and 1.4 for Mn_3_Ga and Mn_2_FeGa, respectively, implying that the tetragonal structure is the ground state for both alloys. Although Mn_3_Ga and Mn_2_FeGa have a local minimum energy at *c*/*a* = 1, we did not observe a cubic state for them in our experiment. It has been reported that, for Mn_2_-based Heusler alloys, when the energy difference (Δ*E*) between cubic and tetragonal structure ≥ 0.1 eV per formula unit (Faleev *et al.*, 2017 ▸), the alloy exists in a stable tetragonal-structure state. The values of Δ*E* for Mn_3_Ga and Mn_2_FeGa are −0.14 and −0.12 eV per formula unit, respectively. This is why only the tetragonal Heusler structure was observed in Mn_3_Ga and Mn_2_FeGa.

**Figure 7:**
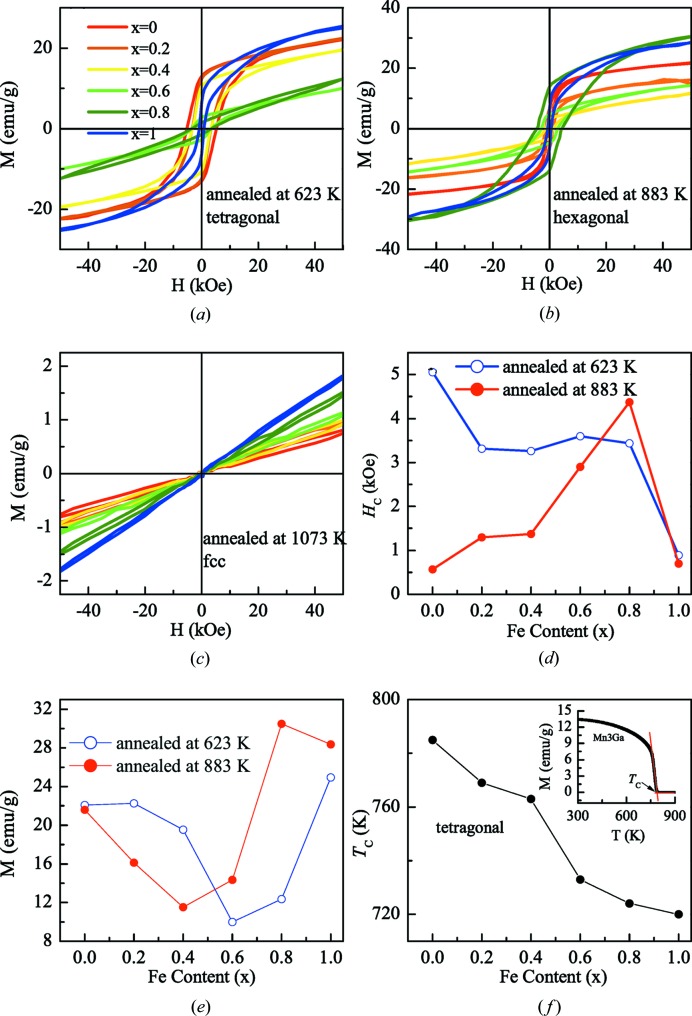
Magnetic hysteresis loops measured at 5 K for Mn_3−*x*_Fe_*x*_Ga samples treated at (*a*) 623 K, (*b*) 883 K and (*c*) 1073 K; (*d*) the coercive field as a function of Fe content for samples treated at 623 and 883 K; (*e*) the magnetization under a field of 50 kOe at 5 K as a function of Fe content; (*f*) the Curie temperature *T*_C_ as a function of Fe content for the tetragonal Mn_3−*x*_Fe_*x*_Ga alloy, and the inset shows the M–T curves of Mn_3_Ga, showing the tangent method for determining *T*_C_.

Different crystal structures result in different magnetic properties of the same alloy. Fig. 7 ▸ shows the magnetic hysteresis curves of Mn_3−*x*_Fe_*x*_Ga samples (*x* = 0, 0.2, 0.4, 0.6, 0.8, 1) at 5 K, with different heat-treatment conditions noted in each figure.

Figs. 7(*a*), 7(*b*) and 7(*c*) show the M–H curves measured at 5 K for Mn_3−*x*_Fe_*x*_Ga (*x* = 0, 0.2, 0.4, 0.6, 0.8, 1) treated at 623, 883, and 1073 K, corresponding to tetragonal, hexagonal and f.c.c. structures, respectively. Owing to the high anisotropy of tetragonal and hexagonal structures, the M–H curves show obvious hysteresis. The magnetization at a high field of 50 kOe (1 Oe = 79.5775 A m^−1^) did not saturate, but for samples with f.c.c. structures (shown in Fig. 7 ▸ *c*), the magnetization increased linearly with the magnetic field, showing a typical antiferromagnetic nature. Felser *et al.* (2013 ▸) reported that an Mn_2_FeGa alloy with an f.c.c. structure is almost in a compensated antiferromagnetic state (Winterlik *et al.*, 2012 ▸).

The coercive field *H*_C_ of the tetragonal structure (sample heat treated at 623 K) decreases from 5 to 0.8 kOe, as Fe content *x* increases from 0 to 1. Namely, *H*_C_ decreases with an increasing *c*/*a* ratio (see Fig. 7 ▸ *d*). Generally, the magnetocrystalline anisotropy increases with an increase in *c*/*a* ratio; however, an opposite trend was observed here. This is likely to be caused by the introduction of Fe content which destroys the coupling of the triangular antiferromagnetism in Mn_3_Ga, resulting in a decrease in anisotropy. According to the law of approach to saturation, the saturation magnetization for Mn_2_FeGa is 16.76 e.m.u. g^−1^, corresponding to 0.71 µ_B_ per formula unit, which is in good agreement with the calculated magnetic moment of 0.80 µ_B_ per formula unit for the stable tetragonal structure. The calculated individual moments of Mn(*a*), Mn(*b*) and Fe are 2.14, −2.60 and 1.26 µ_B_ in Mn_2_FeGa, respectively. The Mn(*a*) and Mn(*b*) atoms have opposite signs of moment, indicating the antiparallel alignment of the atomic moment. This is mainly attributed to the short distance between the nearest neighbouring Mn(*a*) and Mn(*b*) atoms, as observed in most of Mn_2_-based Heusler alloys (Wurmehl *et al.*, 2006 ▸). The magnetization at high field decreases slowly with increasing Fe content initially, reaching its minimum at *x* = 0.6, followed by an increase [summarized in Fig. 7 ▸(*e*)]. This is different from magnetization in tetragonal Mn_3−*x*_Co_*x*_Ga alloys, in which the magnetization decreases monotonously with increasing Co (Felser *et al.*, 2013 ▸).

The Mn-rich tetragonal Heusler alloys usually have high Curie temperatures (*T*_C_), thus we measured the thermomagnetization curves in high-temperature ranges. Fig. 7 ▸(*f*) summarizes the *T*_C_ of tetragonal samples. It was found that *T*_C_ decreases from 785 K in Mn_3_Ga to 720 K in Mn_2_FeGa with increasing Fe content. In tetragonal Mn_3_Ga, one Mn atom resides in the Mn–Ga plane and another two Mn atoms reside in the Mn–Mn plane [see inset (*b*) in Fig. 6 ▸]. The moments of Mn atoms couple antiferromagnetically between these two planes. By introducing Fe into Mn_3_Ga by replacing Mn (*i.e.* Mn_2_FeGa), one Fe atom is located in the Mn–Ga plane and the other resides in the Mn–Fe plane of inverse-tetragonal Mn_2_FeGa. Due to the expansion of the *c* axis with increasing Fe in the tetragonal structure (see Fig. 2 ▸), the distance between the two planes also increases. This results in decreased antiferromagnetic exchange interactions between Mn atoms in these two planes, thus a decrease in *T*_C_. The large coercive field, low magnetization and high *T*_C_ of this tetragonal system suggests that they have potential application in STT-based devices.

Comparing the hexagonal alloys (treated at 883 K), as shown in Fig. 7 ▸(*d*), the coercive field increased initially with increasing Fe content, reaching a maximum value of 4.5 kOe in the Mn_2.2_Fe_0.8_Ga alloy, followed by a rapid decrease to only 0.5 kOe in the hexagonal Mn_2_FeGa alloy. The high-field magnetization of hexagonal Mn_3−*x*_Fe_*x*_Ga decreases first, followed by an increase with increasing Fe content, achieving the minimum at *x* = 0.4 (see Fig. 7 ▸ *e*).

**Figure 8:**
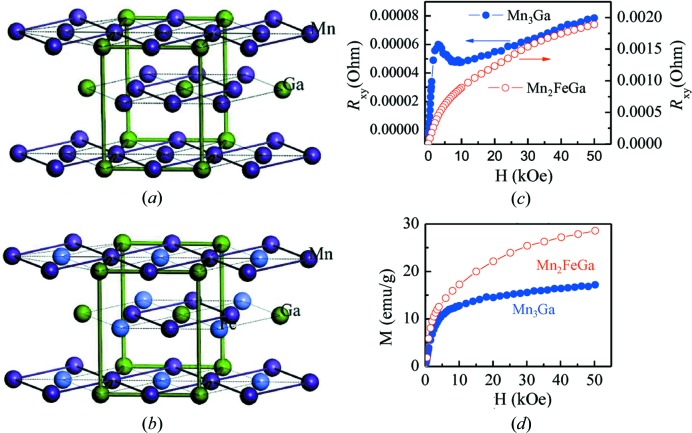
Schematic representation of hexagonal (*a*) Mn_3_Ga and (*b*) Mn_2_FeGa; (*c*) Hall resistance *R*_*xy*_ as a function of the magnetic field measured at 5 K for hexagonal Mn_3_Ga and Mn_2_FeGa alloys; (*d*) initial magnetization curves at 5 K for hexagonal Mn_3_Ga and Mn_2_FeGa alloys.

The crystal structure of hexagonal Mn_3_Ga is shown in Fig. 8 ▸(*a*). By crystallographically introducing Fe atoms into Mn_3_Ga (*i.e.* Mn_2_FeGa), Fe atoms may occupy sites in the same or different layers. There are several possible crystallographic configurations. First-principles calculations suggest Fe atoms prefer to occupy sites in different layers (Kundu & Ghosh, 2017 ▸), as shown in Fig. 8 ▸(*b*). Previously, it has been reported that hexagonal Mn_3_Ga has a non-collinear antiferromagnetic structure with a Neel temperature of *T*_N_ = 460 ± 10 K (Krén & Kádár, 1970 ▸). It was found that there are two types of triangular networks in each plane. The in-plane interactions between Mn atoms are comparable, which ensures a 120° orientation between Mn neighbours. Directions of magnetic moments in the same layer are not equally separated by 120°, therefore they do not fully cancel each other out, resulting in a weak ferromagnetic phase with a small formula moment of 0.03 µ_B_ (Krén & Kádár, 1970 ▸). Recently, first-principles calculations have shown that hexagonal Mn_2_FeGa is a collinear magnetic structure with a magnetic moment of 1.30 µ_B_ (Kundu & Ghosh, 2017 ▸), which is in agreement with our experimental result of 1.26 µ_B_. There are two Mn atoms and one Fe atom in one triangular network, as shown in Fig. 8 ▸(*b*), implying three pairs of exchange interactions in this network. The Fe atom forms ferromagnetic coupling with one Mn and antiferromagnetic coupling with the other Mn, while antiferromagnetic coupling is present between the two Mn atoms (Kundu & Ghosh, 2017 ▸). Competition between these interactions destroys the magnetic frustration in the Mn_3_Ga system and a collinear-like magnetic structure of Mn_2_FeGa is obtained (Kundu & Ghosh, 2017 ▸). Hence, there is a transition from non-collinear magnetism in Mn_3_Ga to collinear spin configurations in Mn_2_FeGa upon the introduction of Fe content. This leads to an initial decrease in saturation magnetization and then an increase with increasing Fe content from *x* = 0 to *x* = 1, as shown in Fig. 7 ▸(*e*).

This magnetic-coupling transition can be also confirmed by Hall resistance. Fig. 8 ▸(*c*) shows Hall resistance *R*_*xy*_ as a function of magnetic field measured at 5 K for hexagonal Mn_3_Ga and Mn_2_FeGa alloys. The shape of the *R*_*xy*_–H curve for Mn_3_Ga is different to that of the M–H curve (Fig. 8 ▸ *d*). A hump-like anomaly for *R*_*xy*_ can be clearly observed for Mn_3_Ga. This hump-like shape is generally considered as a unique symbol of THE (Li *et al.*, 2013 ▸). THE is distinct in prominent non-planar magnetic configurations (Sürgers *et al.*, 2014 ▸), further indicating our Mn_3_Ga sample possesses non-collinear magnetism. In comparison, for the Mn_2_FeGa alloy, a hump-like shape is absent. The Hall resistivity increases with magnetic field and no saturation is achieved, exhibiting the same behaviour shown in the M–H curve (see Fig. 8 ▸ *d*), which suggests no additional contribution of THE. These results demonstrate that the non-collinear magnetic structure is present in Mn_3_Ga and collinear spin configurations form in Mn_2_FeGa. The rich magnetic properties of hexagonal Mn_3−*x*_Fe_*x*_Ga alloys, including the non-collinear and collinear magnetism, result in many useful functional properties, *i.e.* the topological Hall effect in Mn_3_Ga and exchange bias in Mn_2_FeGa (Liu *et al.*, 2016 ▸). This means these alloys possess great potential for technological applications.

Based on the above discussions, we can see that the crystal structures and magnetic properties of Mn_3−*x*_Fe_*x*_Ga alloys (*x* = 0, 0.2, 0.4, 0.6, 0.8, 1) can be extensively tailored by different heat-treatment conditions. The multiple crystal structures may be caused by magnetism. It has been reported that the crystal structures of many alloys and compounds are sensitive to magnetism (Söderlind & Moore, 2008 ▸). If the energy differences between different structures is small, a strong magnetic moment may overcome the energy difference between the different crystal structures, which will, in turn, influence the crystal structures (Söderlind & Moore, 2008 ▸). Because of the diverse structures and properties of these materials, this work can therefore offer a way to design new multifunctional materials towards spintronic applications.

## 4. Conclusions   

This study investigated the structural and magnetic properties of Mn_3−*x*_Fe_*x*_Ga alloys (*x* = 0, 0.2, 0.4, 0.6, 0.8, 1) under different heat-treatment conditions. Tetragonal alloys were observed after heat treatment at 623 K for three days followed by quenching in water. These tetragonal alloys present a large coercive field and low magnetization. Hexagonal alloys were produced after heat treatment at 883 K. A decrease of coercive field was observed for the hexagonal alloys. Mn_3−*x*_Fe_*x*_Ga alloys with a hexagonal structure, showing non-collinear and collinear magnetic properties, exhibit distinct properties such as the topological Hall effect. The same alloys crystallized in f.c.c. lattices after heat treatment at 1073 K. In contrast to the tetragonal and hexagonal structures, the alloys with an f.c.c. structure are antiferromagnetic in nature. The isomeric Mn_3−*x*_Fe_*x*_Ga materials have various spin structures, which enable them to exhibit rich physical properties.
